# William Harry Hannon—A Life Well Lived

**DOI:** 10.3390/ijns8020037

**Published:** 2022-06-17

**Authors:** Bradford L. Therrell, Robert F. Vogt, Joanne V. Mei

**Affiliations:** 1 Department of Pediatrics, University of Texas Health Science Center at San Antonio, San Antonio, TX 78229, USA; 2 National Newborn Screening and Global Resource Center, Austin, TX 78759, USA; 3 Newborn Screening and Molecular Biology Branch, Centers for Disease Control and Prevention, Atlanta, GA 30329, USA; rvogt@cdc.gov (R.F.V.); jvm0@cdc.gov (J.V.M.)

Dr. William Harry Hannon (Harry) (Figure 1), Buford, Georgia found eternal peace on Friday, 6 May 2022, at Northeast Georgia Medical Center in Braselton, Georgia, USA. Harry was preceded in death by his parents James Henry and Jeanette Bentley Hannon of Covington, Georgia. He is survived by his daughter, Terri Fain; son, John Hannon; brother and sister-in-law, James H. (Jimmy) and Lynn Hannon, Jr.; sisters and brothers-in-law, Sandra and Gerald Yates, and Margaret and Mike Burgess; sister, Starr Strickland; grandchildren, Spencer Cape, Zachary Thomas, Shelby Opperman, Joseph Hannon, Austin Fain, and Katherine Fain; and three great-grandchildren.

He was preceded in death by his beloved wife, Barbara Cheryl Hannon (Cherry).

In retirement, Harry enjoyed spending time with his children and grandchildren. He continued his work in the field of public health newborn screening (NBS), initiating, expanding, and improving NBS worldwide.

Born 9 June 1941, in Covington, Georgia, Dr. W. Harry Hannon lived an outstanding life that included many significant and lasting contributions to the advancement in public health laboratory science. Harry graduated from Tucker High School in 1959, received his B.S. in Chemistry from Georgia State University in 1965, and his PhD in Biochemistry from the University of Tennessee in 1972. He completed his formal education in 1974 with post-doctoral training at the Oak Ridge National Laboratories where he used novel methods that gave rise to the field of proteomics. His areas of expertise included immunochemistry, dried-blood spot technologies, NBS for metabolic disorders, and laboratory quality assurance systems. In 2009, Harry retired from the CDC with 41 years of federal service, having spent over 25 years as the Chief of what is now known as the Newborn Screening and Molecular Biology Branch |CDC.

It would be difficult to overstate the impact of Harry’s work on public health NBS. He authored or co-authored more than 250 scientific publications and served on over 30 national and international committees addressing various newborn laboratory issues. Harry was careful not to overstep his knowledge or experience in answering requests for help and often included others with more relevant experiences as co-authors or co-committee members when addressing such requests. Examples of his inclusionary efforts include such items as guideline booklets prepared for World Health Organization defining procedures useful in developing countries for implementing screening for both phenylketonuria and congenital hypothyroidism (published in 1990 and 1991, respectively), and 14 book chapters on topics such as laboratory methods for detecting congenital hypothyroidism (1993) and congenital hypothyroidism (2000), and an overview of the history and applications of dried-blood samples (2014).

Of particular importance were improvements in harmonizing and standardizing NBS methods. Chief among his accomplishments was his response to a request from Dr. Robert Guthrie in early 1979, to create a national NBS quality control program at the CDC (see Figure 2). Under Dr. Hannon’s direction, the CDC’s Newborn Quality Assurance Program (NSQAP) became an integral part of the NBS systems in the United States and globally. The NSQAP, by providing proficiency testing, training, reference materials, and consultative services, serves as a center of expertise for all state NBS laboratories and approximately 670 NBS laboratories throughout the United States and 87 other countries. These activities have included working with commercial kit manufacturers and professional organizations with similar interests.

In 1981, in collaboration with the Texas Newborn Screening Laboratory, Harry assisted in founding the first U.S. National NBS Symposium (today, the APHL-sponsored Newborn Screening and Genetic Testing Symposium). Because he was interested in international quality assurance (QA) issues in NBS, he attended the first international NBS QA meeting in Japan in 1987. The International Society for Neonatal Screening (ISNS) was organized at this meeting. Over the following years, Harry would become an active ISNS member serving on the ISNS International QA Committee, the ISNS Council (1999–2002), as ISNS Vice-President (2002–2009), and as a member of the Editorial Committee of the journal *Screening* (the ISNS journal at the time). He received the *ISNS-Robert Guthrie Award* in 1999 in “Worldwide recognition of outstanding contributions to newborn screening”, and was elected as an ISNS Honorary Member in 2009. Additionally, in 1987, the U.S. Health Resources and Services Administration organized a National NBS Review Team to review and improve U.S. NBS Programs (34 reviews completed). He was also active as a proposal reviewer for the CDC Foundation’s NBS Translational Research Initiative.

Dr. Hannon had an outstanding 41-year career at the CDC that included receipt of over 35 special recognition and service awards. He was awarded the CDC’s highest honor for scientific excellence, the *Charles C. Shepard Science Award* in 1992 and again in 2005 (see Figure 3). In 2006, he was awarded CDC Sigma Xi’s *Walter Dowdle Award* for “Achievements in Public Health Laboratory Science” and in 2008, the Association of Public Health Laboratories presented him with their *Lifetime Achievement Award* for “Leadership in the field of public health laboratory science and influencing public health policy on a national and global level.” Additionally, the APHL created a global NBS award, *The Harry Hannon Laboratory Quality Improvement Award,* to be presented at each U.S. national NBS meeting. Harry was also involved with parent support activities serving as a Board Member of several such groups. In April 2009, he received the Jeffrey Modell Foundation’s *Dream Makers Award* as a “Pioneer in Newborn Screening” for contributions to the early detection of severe combined immunodeficiency disorders (SCIDs) by NBS.

His committee work with the Clinical Laboratory Standards Institute (CLSI) was instrumental in setting CLSI standards and guidelines for national and international health laboratory practice. Harry chaired the working groups on the first seven approved editions of the only “standard” specifically targeted at NBS, *NBS 01: Blood Collection on Filter Paper for Newborn Screening*. Harry’s vision for laboratory quality in NBS seeded the development of 13 CLSI standards/guidelines that have proven to be invaluable for NBS professionals worldwide. In 2008, CLSI awarded Harry its *Russell J. Eilers Award* (CLSI’s highest award) for outstanding contributions in developing clinical laboratory standards.

See Figure 4 for images of Dr. Hannon across his career. His contributions to NBS and public health will not soon be forgotten, and his accomplishments will stand for many years in testimony of a life well lived!

## Figures and Tables

**Figure 1:**
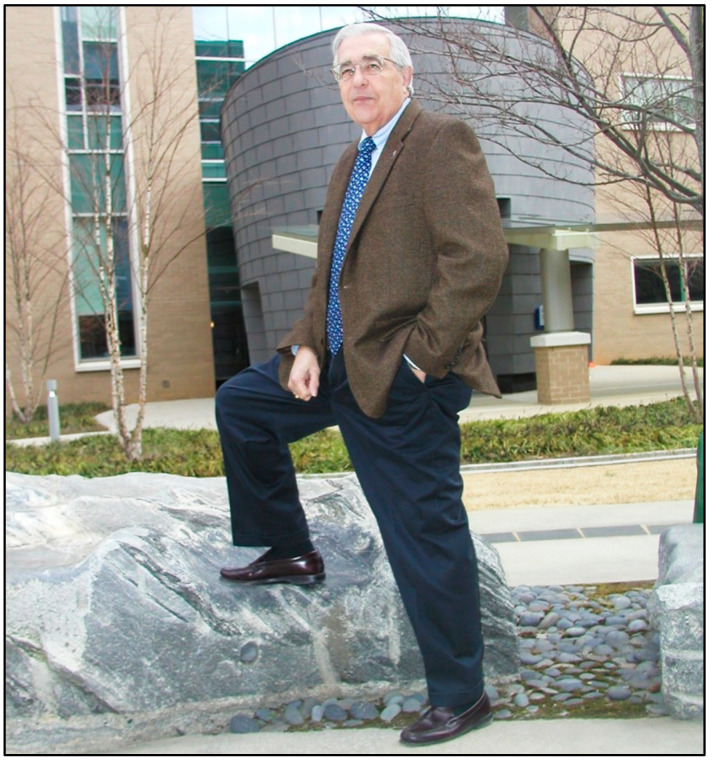
Dr. William Harry Hannon (Provided by Hannon family friend).

**Figure 2:**
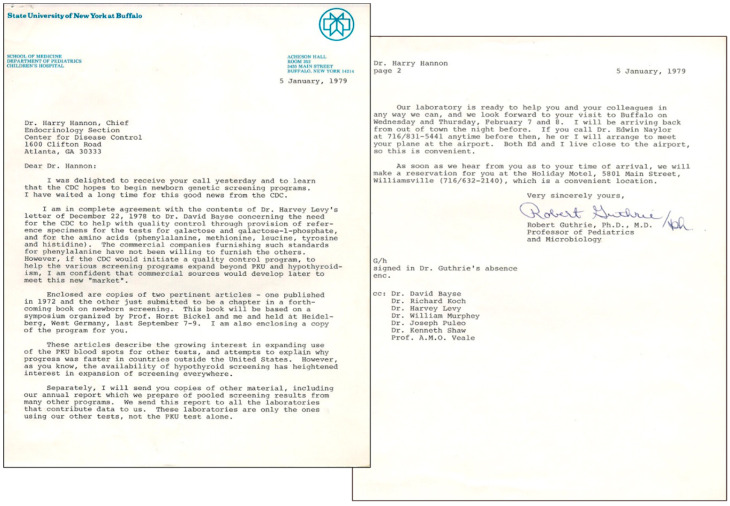
Personal letter to Dr. Hannon from Dr. Robert Guthrie in 1979 regarding CDC activities.

**Figure 3:**
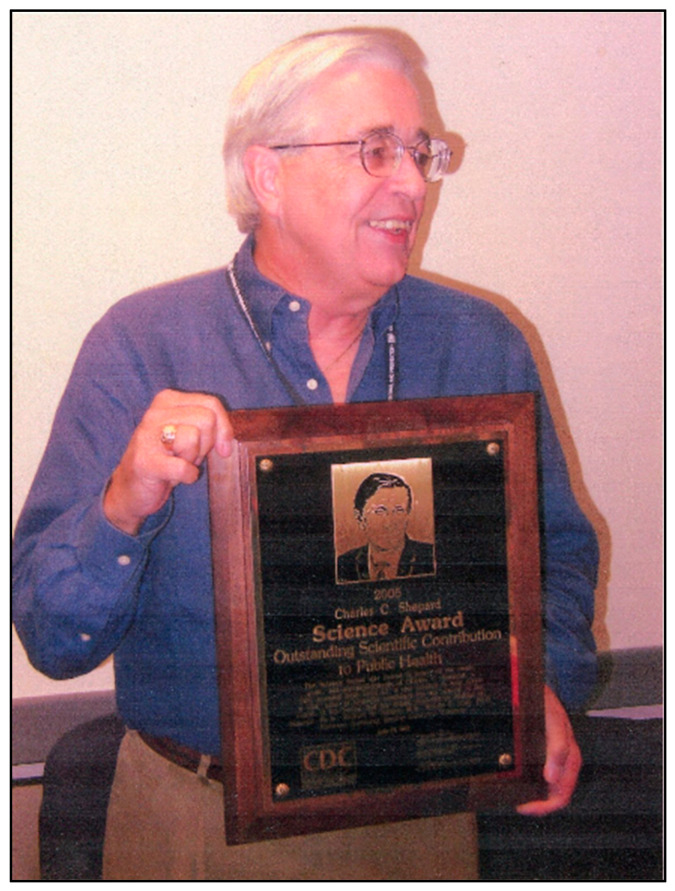
Dr. Hannon with 2005 Shepherd Award. (Photo provided by and approved for use by CDC.).

**Figure 4:**
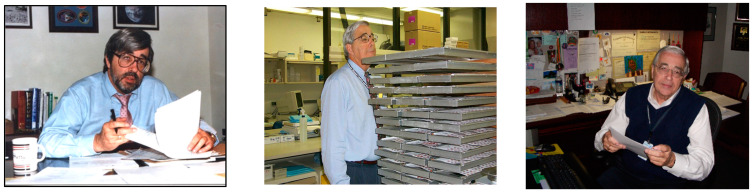
Various pictures over Dr. Hannon’s career. Picture on left shows Dr. Hannon in 1988, with more recent pictures center and right. (All photos donated by Hannon family friend for this publication.).

